# The COVID-19 pandemic did not negatively impact frequency or continuity of outpatient care in Alberta, Canada

**DOI:** 10.1038/s41598-023-43064-3

**Published:** 2023-09-21

**Authors:** Finlay A. McAlister, Zoe Hsu, Yuan Dong, Erik Youngson

**Affiliations:** 1 The Division of General Internal Medicine, Faculty of Medicine and Dentistry, University of Alberta, 5-134C Clinical Sciences Building, 11350 83 Avenue, Edmonton, AB T6G 2G3 Canada; 2 The Alberta Strategy for Patient Oriented Research Support Unit, Edmonton, Canada; 3 Provincial Research Data Services, Alberta Health Services, Edmonton, Canada

**Keywords:** Health care, Health services, Viral infection

## Abstract

Outpatient care patterns have changed markedly during the COVID-19 pandemic. In this population-based retrospective cohort study, we compared the frequency of outpatient care (whether in-person or virtual) and continuity of care for all community-dwelling adults in Alberta between March 1, 2019 and February 29, 2020 (pre-pandemic) versus March 1, 2020 to February 28, 2021 (pandemic). We calculated provider continuity using Breslau’s Usual Provider Continuity (UPC) for patients with at least 2 outpatient encounters. In 2019–20, 594,350 (98.4%) of 603,877 community-dwelling adults with ambulatory care sensitive conditions (ACSC) had $\ge$ 1 outpatient visit (median 8 visits, mean UPC score 0.61, SD 0.23), compared to 566,569 (98.6%) of 574,613 (median 8 visits, mean UPC score 0.67, SD 0.23) during the first year of the pandemic. Similar patterns were seen for adults without ACSC: 2,207,710 (93.9%) of 2,350,147 had $\ge$ 1 outpatient visit (median 3 visits, mean UPC score 0.61, SD 0.24) pre-pandemic compared to 2,113,239 (93.5%, median 4 visits, mean UPC 0.67, SD 0.24) in the first year of the pandemic. Thus, the COVID-19 pandemic did not impact frequency of follow-up while continuity of care improved both for patients with or without ACSC in Alberta, Canada.

## Introduction

Continuity of care in the outpatient setting is associated with greater patient satisfaction, increased likelihood of receiving guideline-recommended care, and better outcomes, with the greatest benefits seen in those with chronic conditions^1–3^. This is particularly important for patients with ambulatory care sensitive conditions (ACSC), defined by the Canadian Institute of Health Information as asthma, chronic obstructive pulmonary disease, heart failure, coronary disease, hypertension, diabetes, and epilepsy. However, the impact of the COVID-19 pandemic, and particularly the marked increase in virtual visits in lieu of in-person visits during the pandemic documented in various countries^4–7^, on physician continuity of care is unknown.

During the early stages of the COVID-19 pandemic, a stay-at-home lockdown with closure of all non-essential businesses and services was initiated in Alberta, Canada from March 15, 2020 to June 15, 2020. Although physician offices were not explicitly asked to close down, a variety of public health restrictions were in place in Alberta on and off until June 14, 2022 and physicians were asked to minimize in-person outpatient encounters as much as possible, particularly in the first year of the pandemic. To facilitate this, on March 17, 2020 the provincial government (the single payer for healthcare in Alberta) introduced new physician billing codes for virtual (telephone or video) outpatient visits that were set at the same remuneration level as for in-person outpatient encounters. We recently reported that in-person outpatient physician visits for Albertan adults declined by 39% in the first year of the pandemic, but the increase in virtual visits (the vast majority of which were telephone-based) more than offset this reduction such that total outpatient encounters increased by 4% in that year^8^. We also found that the proportion of community-dwelling Albertan adults presenting to an emergency department at least once decreased (from 40.1 to 34.3% for those with ACSC and from 25.5 to 22.3% for those without ACSC), as did the proportion requiring hospitalization (from 16.2 to 14.8% in those with ACSC and from 5.5 to 5.3% in those without ACSC)^8^. We found that the COVID-19 pandemic did not negatively impact the frequency of outpatient follow-up, prescribing, or 3 month outcomes for adults with heart failure, diabetes mellitus, or hypertension^8^. However, in that manuscript we did not explore the impact of the pandemic on the broader population of adults with non-cardiovascular ACSC or adults without ACSC, nor its impact on continuity of care in any patient group.

The aims of this study were to describe the impact of the pandemic on outpatient care patterns and physician continuity of care for all adults in Alberta, Canada and comparing whether any changes were similar or different in those with versus without ACSC. We also wanted to describe any changes in medication dispensations for adults with or without ACSC in Alberta.

## Methods

### Study design

This was a retrospective cohort study of all adult Albertans using physician services in two sequential time periods: March 1 2019 to February 29 2020 (classified as ‘pre-pandemic’); and March 1, 2020 to February 28, 2021 (first year of the pandemic).

### Data sources and study sample

The Canadian province of Alberta has a publicly funded health care system with universal access and without user fees at the point of care. This study deterministically linked several healthcare datasets in for all non-institutionalized adult Albertans. The Healthcare Practitioner Claims Database captures all physician visits anywhere in the province (including those shadow-billed by salaried physicians), includes the date and up to 3 diagnoses for each visit, and after March 17, 2020 included new codes to identify virtual (telephone or video) visits. We used the information from the Discharge Abstract Database (which captures the dates and up to 25 diagnoses and 10 procedures for all acute care hospitalizations) and the Ambulatory Care Database (which captures the dates date and up to 10 diagnostic codes for all Emergency Department assessments and hospital-based physician office visits) to build comorbidity profiles for each patient. The Pharmacy Information Network (PIN) captures the dates of all medication dispensations from community pharmacies. The Alberta Health Care Insurance Registry captures patient demographics, including home addresses. The comprehensiveness and accuracy of the databases we used in this study have been previously established^9^.

### Continuity measure

We used the Healthcare Practitioner Claims Database to identify all physicians (primary care physicians and specialists) each Albertan adult saw and identified the physician they saw most often in each study time period. We calculated provider continuity using Breslau’s Usual Provider Continuity (UPC), calculated as n/N, where n is the number of visits to their most commonly seen physician and N is the total number of visits to any physician in that timeframe, with UPC scores ranging from 0 (perfect “discontinuity”) to 1 (perfect continuity)^10^. Patients who died or left the province were censored at the date of death or emigration (this is an uncommon phenomenon, in the year prior to the pandemic, 0.14% of all Albertan adults emigrated and 0.76% died; in the first year of the pandemic the corresponding proportions were 0.11% and 0.77%). Of note, we treated virtual or in-person visits the same for the purposes of calculating UPC (that is, both were defined as outpatient encounters) and patients had to have had at least two outpatient encounters in either timeframe to contribute to the UPC analysis.

### Comorbidities

We used ICD-9 and ICD-10-CA case definitions previously validated in Alberta for any hospitalizations, any ED visits, and any outpatient visits in the 2 years prior to and including the index visit to identify comorbidities for each patient^9^. We used previously validated case definitions to identify those patients with at least one ACSC (eAppendix Table 1)^11^.

### Statistical analysis

We report care patterns and UPC by presence/absence of ACSC. Differences were assessed for statistical significance (*p* < 0.05) using the Chi-square test or the Mann–Whitney U test/Wilcoxon rank-sum test as appropriate. In a sensitivity analysis for only those patients receiving outpatient care in both the pre-pandemic and pandemic years, we examined whether their most common provider in the pandemic year was the same as in the pre-pandemic year. All statistical analyses were done using SAS v.9.4 (Cary, North Carolina)^12^.

### Ethics

The University of Alberta Health Research Ethics Board approved this study (Pro00115481) and the University of Alberta Health Research Ethics Board waived the need for individual patient informed consent since the investigators were only provided with de-identified data. Our work was performed in accordance with the Declaration of Helsinki and was reported as recommended by the Strengthening the Reporting of Observational Studies in Epidemiology (STROBE) guidelines^13^.

## Results

In 2019–20, 594,350 (98.4%) of 603,877 community-dwelling adults with ACSC had at least one outpatient visit (median 8 visits), compared to 566,569 (98.6%) of 574,613 (median 8 visits) during the first year of the pandemic (Table 1). Similar patterns were seen for adults without ACSC: 2,207,710 (93.9%) of 2,350,147 had at least one outpatient visit (median 3 visits) pre-pandemic compared to 2,113,239 (93.5%, median 4 visits) in the first year of the pandemic. Mean UPC scores for community-dwelling adults having two or more outpatient visits in the year prior to the pandemic were 0.61 (SD 0.23) for those with ACSC and 0.61 (SD 0.24) for those without ACSC, compared to 0.67 (SD 0.23) in ACSC patients and 0.67 (SD 0.24) in those without ACSC during the first year of the pandemic (Table 1, both *p* < 0.001 comparing pre- with pandemic UPC scores for adults with or without ACSC). In our sensitivity analysis of the 1,758,540 patients receiving at least 2 outpatient visits in both the pre- and pandemic year, we found that the most commonly seen provider was the same in both time periods for 74% of patients with ACSC and 64% of patients without ACSC.

**Table 1:** Health service use by community dwelling adults in Alberta in the year prior to and the first year of the COVID-19 pandemic.

	Year prior to pandemic			First year of pandemic		
	Overall	No ACSC	≥ 1 ACSC	Overall	No ACSC	≥ 1 ACSC
Number	2,954,024	2,350,147	603,877	2,834,768	2,260,155	574,613
Median age (IQR)	46 (33, 60)	41 (30, 56)	62 (50, 72)	47 (33, 61)	42 (31, 57)	62 (51, 72)
Female %	1,566,278 (53.0)	1,276,491 (54.3)	289,787 (48.0)	1,521,112 (53.7)	1,245,966 (55.1)	275,146 (47.9)
Median charlson score	0 (0, 0)	0 (0, 0)	1 (0, 1)	0 (0, 0)	0 (0, 0)	1 (0, 1)
Urban residence	2,564,063 (86.8)	2,052,646 (87.3)	511,417 (84.7)	2,462,677 (86.9)	1,972,893 (87.3)	489,784 (85.2)
Healthcare encounters						
Had at least one outpatient encounter (any physician, in person or virtual)	2,802,060 (94.9)	2,207,710 (93.9)	594,350 (98.4)	2,679,808 (94.5)	2,113,239 (93.5)	566,569 (98.6)
Median number of outpatient encounters (any physician, in person or virtual)	4 (2, 8)	3 (2, 6)	8 (5, 12)	4 (2, 8)	4 (2, 7)	8 (4, 13)
Saw PCP in person	2,733,529 (92.5)	2,146,521 (91.3)	587,008 (97.2)	2,297,134 (81.0)	1,785,902 (79.0)	511,232 (89.0)
Median number of PCP in person visits	3 (2, 6)	3 (1, 5)	6 (4, 9)	2 (1, 4)	2 (1, 3)	3 (1, 6)
Virtual visit with PCP	0 (0.0)	0 (0.0)	0 (0.0)	1,703,908 (60.1)	1,260,359 (55.8)	443,549 (77.2)
Median number of PCP virtual visits	0 (0.0)	0 (0.0)	0 (0.0)	1 (0, 3)	1 (0, 2)	2 (1, 5)
Had at least one medication dispensation	2,525,365 (85.5)	1,930,374 (82.1)	594,991 (98.5)	2,393,089 (84.4)	1,826,683 (80.8)	566,406 (98.6)
Median number of medications dispensed	4 (1, 8)	3 (1, 6)	9 (5, 14)	3 (1, 7)	3 (1, 6)	8 (5, 13)
Had at least one ED visit	841,137 (28.5)	599,029 (25.5)	242,108 (40.1)	701,685 (24.8)	504,460 (22.3)	197,225 (34.3)
Had at least one hospitalization	227,059 (7.7)	129,308 (5.5)	97,751 (16.2)	205,805 (7.3)	120,605 (5.3)	85,200 (14.8)
Had at least 2 outpatient encounters (any physician, in person or virtual)	2,390,926 (80.9)	1,808,062 (76.9)	582,864 (96.5)	2,274,661 (80.2)	1,720,042 (76.1)	554,619 (96.5)
Usual Provider Continuity (SD)	0.61 (0.23)	0.61 (0.24)	0.61 (0.23)	0.67 (0.24)	0.67 (0.24)	0.67 (0.23)

Data reported as counts and percentages.

*ACSC* ambulatory care sensitive condition, *PCP* primary care physician, *ED* emergency department, *IQR* interquartile range.

In 2019–20, 594,991 (98.5%) of 603,877 community-dwelling adults with ACSC received at least one prescription and the median number of prescriptions dispensed was 9, while in the first year of the pandemic 566,406 (98.6%) of 574,613 received at least one prescription and the median number of prescriptions was 8 (Table 1). Similar patterns were seen for adults without ACSC: 1,930,374 (82.1%) of 2,350,147 had at least one prescription (median 3) pre-pandemic compared to 1,826,683 (80.8%, median 3) of 2,260,155 in the first year of the pandemic.

## Interpretation

We found that the COVID-19 pandemic was not associated with changes in the frequency of outpatient follow-up or medication dispensations but was associated with improved continuity of care both for patients with or without ACSC in Alberta, Canada.

Our finding that frequency of followup and medication dispensations for patients with ACSC were stable during the first year of the pandemic provides reassurance that access to outpatient care for patients with chronic conditions was not adversely impacted by the pandemic, at least in our province where the government funder remunerated virtual visits at the same level as in-person visits and enabled a rapid shift towards a mixed in-person/virtual model for outpatient care. Our finding that care and prescribing patterns were also very similar after pandemic onset compared to the prior year in patients without ACSC also provides some reassurance that although earlier studies reported marked reductions in outpatient visits in the first few months of the pandemic^4,5^, this had largely recovered by the end of the first year, at least in our province. Our findings also call into question the commonly-held assumption expressed by two thirds of healthcare policy makers and administrators^14^ and one third of elderly Americans^15^ in recently published surveys that access to outpatient care for non-COVID conditions deteriorated after onset of the pandemic. Interestingly, we found that provider continuity actually improved for both those with or without ACSC during the pandemic, likely reflecting the fact that virtual care is easier to implement with patients already known to physicians rather than for new patients.

However, as we don’t have any data on the content of each outpatient encounter, we are unable to address the question of whether quality and outcomes of outpatient care deteriorated after pandemic onset, two other concerns raised in the surveys of elderly Americans and healthcare administrators^14,15^. Further research is needed to investigate whether the shift towards virtual outpatient care has negatively impacted screening for and detection of new conditions (such as diabetes, atrial fibrillation, or cancer). For example, a recent US study reported that the frequency with which six established primary care screening quality measures were done declined by 2/3 in the early months of the pandemic and was still 1/3 lower when COVID-19 cases had declined markedly between the first and second waves^16^. Unfortunately, there are also now multiple analyses demonstrating declines in new cancer diagnoses and worsening prognosis for those detected (presumably later in their disease course)^17–20^ as well as poorer maternal and fetal outcomes^21^ during the COVID pandemic.

Our finding that outpatient care shifted quickly after the onset of the COVID-19 pandemic to a mixed model incorporating both in-person and virtual visits mirrors reports from other locales. However, while those studies focused on selected populations, selected diagnoses, selected visits (primary care only), or only examined data from the first 3–4 months of the pandemic^4–7,22,23^, we were able to examine all adult outpatient visits (primary care and specialty) for any cause in an entire Canadian province over the first year of the pandemic, which included the lulls between COVID-19 waves. While we did not have data on type of virtual care, a report from the Canadian Institute of Health Information confirmed that over 92% of virtual visits in 2021 were telephone conversations between patient and physician (2% were videoconferences and 6% were text exchanges)^24^.

While this study includes data from an entire population in a universal access, single payer health care system, there are some limitations to our data. For one, the generalizability of our findings to other health care systems are uncertain as there are likely to be substantial variations in the use of virtual visits between geographic regions reflecting local administrative and physician remuneration policies (not all jurisdictions remunerated virtual visits at the same level as in-person visits during the pandemic). Further, US investigators have reported substantially lower use of virtual visits by individuals living in socially disadvantaged neighbourhoods, raising concerns about virtual care accentuating healthcare disparities^5,25^. Regardless, our data at least shows that in a jurisdiction where physician funding was initiated to enable virtual visits and where healthcare is universal and free at the point of service, there were no negative impacts of the pandemic on access to outpatient care. However, as we did not have data on reasons for, nor content of, visits we could not assess the appropriateness, quality, or cost-effectiveness of outpatient visits before or during the pandemic. Some studies have reported that patients were less likely to have blood pressures or cholesterol measured or medications intensified during virtual visits than in-person visits^5,16,26^, raising the question of whether both types of outpatient encounters (virtual versus in-person) can truly be considered equivalent.

In conclusion, our data provides reassurance that the shifts in outpatient care patterns caused by the COVID-19 pandemic (towards a mix of virtual and in-person visits at the discretion of providers) did not negatively impact frequency of follow-up, continuity, or prescribing for adults, whether or not they had ACSC. However, further research is needed to determine whether quality and outcomes (including patient and provider reported experience measures) are the same for virtual outpatient encounters as for in-person encounters and to define the optimal frequency of each.

### Supplementary Information

Supplementary Information.

## Data Availability

To comply with Alberta’s Health Information Act, the dataset used for this study cannot be made publicly available. The dataset from this study is held securely in coded form within the AbSPORU (Alberta Support for Patient Oriented Research Unit) Data Platform. While legal data sharing agreements between the investigators, AbSPORU, and Alberta Health Services/Alberta Health prohibit us from making the dataset publicly available, access may be granted to those who meet pre-specified criteria for confidential access, available at www.absporu.ca. The dataset analytic codes are available from the corresponding author (McAlister) upon request, understanding that the computer programs may rely upon coding templates or macros that are unique to AbSPORU.
