# Supplementary material for: The COVID-19 pandemic did not negatively impact frequency or continuity of outpatient care in Alberta, Canada
Source: Sci Rep. 2023 Sep 21;13:15691. doi: 10.1038/s41598-023-43064-3 (PMC10514193; doi:10.1038/s41598-023-43064-3)
Supplement: Supplementary file 1 — Supplementary Information. [file 41598_2023_43064_MOESM1_ESM.docx]

**eAppendix Table 1: List of case definitions for ambulatory care sensitive conditions**

Defined by 1 hospitalization or 1 Emergency Department visit or 2 Practitioner Claims within one year.

- **Chronic obstructive pulmonary diseases (COPD)
  ICD-9-CM**: 416, 490, 491, 492, 493, 494, 495, 496, 500, 501, 502, 503, 504, 505 **ICD-10-CA:**J40, J41, J42, J43, J44, J45, J46, J47, J60, J61, J62, J63, J64, J65, J66, J67, I278, I279, J684, J701, J703
- **Asthma
  ICD-9-CM:** 493 **ICD-10-CA:** J45
- **Diabetes
  ICD-10-CA:** E100-E149
- **Epilepsy
  ICD-9-CM:** 345 **ICD-10-CA:** G40, G41
- **Heart failure**

**ICD-9-CM:** 428, 518 **ICD-10-CA:** I50, J81

- **Hypertension
  ICD-9-CM:** 401, 402, 403, 404, 405 **ICD-10-CA:** I10, I11, I12, I13, I15
- **Coronary Disease (Angina)
  ICD-9-CM:** 411, 413 **ICD-10-CA:** I20, I23, I24
